# Histological Processing of Organoids for Immunostaining

**DOI:** 10.21769/BioProtoc.5750

**Published:** 2026-07-20

**Authors:** Lisa Brossard, Victor Perreaux, Simon Vales, Lola Bonneau, Sarah Godin, Anne Bibonne, Theo Noël, Laura Bachir, Archie Khan, Guillaume Lamirault, Anne Gaignerie, Nathalie Gaborit, Maxime M. Mahe

**Affiliations:** 1 Nantes Université, CHU Nantes, Inserm, TENS, The Enteric Nervous System in Gut and Brain Diseases, IMAD, Nantes, France; 2 Nantes Université, CHU Nantes, CNRS, INSERM, l’institut du thorax, Nantes, France; 3 Nantes Université, CHU Nantes, CNRS, Inserm, BioCore, US16, SFR Bonamy, Nantes, France; 4 Center for Stem Cell and Organoid Medicine, Cincinnati Children’s Hospital Medical Center, Cincinnati, OH, USA

**Keywords:** Paraffin embedding, Organoids, HistoGel, Histology, Immunofluorescence, FFPE, Tissue processing

## Abstract

Organoids are three-dimensional cell structures derived from stem cells that recapitulate the architecture and function of native tissues. Histological analysis of organoids is essential for assessing their structure, cellular composition, and responses to experimental conditions. However, their small size and fragility make standard paraffin embedding workflows difficult. Here, we describe a robust and reproducible protocol for the fixation, paraffin embedding, and sectioning of human organoids, enabling high-quality histological and immunostaining analysis. The method involves direct fixation within the culture matrix and inclusion in HistoGel to prevent organoid loss during processing. The protocol is compatible with hematoxylin–eosin (H&E) staining and multiplex immunofluorescence. Critical steps, troubleshooting, and adaptations for intestinal and cardiac organoids are discussed. This cost-effective and accessible method supports long-term preservation and detailed structural analysis of organoid models.

Key features

• Enables reliable fixation, embedding, and sectioning of fragile human organoids for histological and immunofluorescence analysis.

• Uses HistoGel inclusion to prevent organoid loss during paraffin embedding and processing.

• Compatible with multiple organoid types (intestinal, cardiac, etc.) and staining methods (H&E, immunofluorescence).

• Produces high-quality paraffin blocks and sections suitable for long-term storage.

## Graphical overview


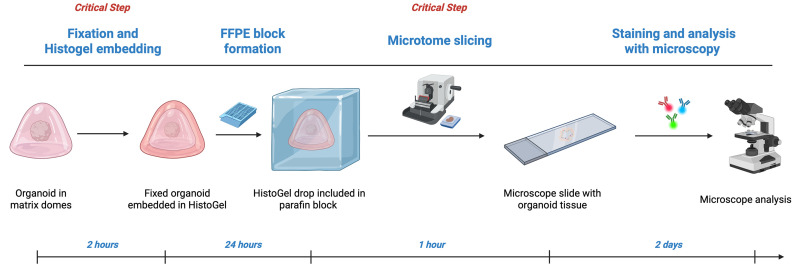


## Background

**Organoid histology:** Organoids are in-vitro 3D cell structures generated from human pluripotent or adult stem cells. Both methods depend on the expansion and differentiation abilities of stem cells to create organ-specific, self-organized structures [1]. The key advantage of organoids is their cellular diversity, architecture, and functions, which resemble those of in vivo organs. Consequently, organoids have become highly valuable for modeling tissue development, adult physiology, and diseases [2]. Additionally, organoids hold significant potential for clinical uses such as personalized medicine, drug screening, and cell therapy [3]. Laboratory analysis of organoids typically involves histological techniques, which can be performed in various ways. One standard method is paraffin embedding, where tissue is fixed, dehydrated, and sliced into thin sections after being embedded in paraffin [4]. Paraffin embedding enables the long-term storage of organoids with minimal tissue degradation and is compatible with techniques such as immunohistochemistry or immunofluorescence. However, due to their limited size, ranging from a few hundred micrometers to a few millimeters, handling, processing, and embedding individual organoids in paraffin is challenging. In this protocol, we outline a versatile method for processing and embedding organoids in paraffin wax for histological analysis. This method requires only basic histology skills for tissue processing and staining. We validated the efficiency and robustness of this technique by applying it to two types of human organoids: intestinal organoids and cardiac ventricular chamber-specific organoids, called cardioids [5,6]. This approach, which involves direct fixation of organoids within their culture matrix combined with the use of a HistoGel, ensures efficient inclusion of organoids in paraffin without risk of loss during processing. Additionally, we describe various staining protocols applicable to different types of organoids.

**Comparison with other methods**: Histological analysis of in vitro organoids can be performed using various methods, each with its own advantages and limitations. Whole-mount staining offers a quick and efficient way to analyze organoids as a whole, while maintaining their three-dimensional structure. This technique is particularly useful for obtaining 3D volume rendering images [7,8]. However, whole-mount staining also presents several technical challenges. Direct whole-mount staining of organoids in their culture matrix can cause excessive background fluorescence and artifacts, while removing organoids from the culture matrix may result in loss of organoids during staining because of their small size. Whole-mount staining also often requires clearing methods, some of which use solvents incompatible with certain antibodies [9]. This technique limits the number of stainings that can be performed on the same organoid. Moreover, colorimetric staining, such as hematoxylin–eosin, cannot be used for whole-mount staining, although some alternatives have been developed [10]. Tissue sectioning is another technique used for organoid histological analysis. This method is especially useful for examining tissues at a sub-cellular level and allows for multiple stains on the same organoid across different sections. Two approaches can be employed: frozen tissue sections and paraffin-embedded sections. Frozen tissue sections involve quickly freezing the organoids, embedding them in a medium like OCT, and then sectioning them with a cryostat before staining. Although frozen sections better preserve the natural protein structure of antigens, they may cause tissue morphology to degrade due to freezing artifacts. The paraffin-embedded section consists of fixing and dehydrating tissue samples before embedding them in paraffin wax and sectioning with a microtome. The main benefit of paraffin sections is their excellent preservation of tissue morphology, along with cost-effective storage for indefinite periods. Additionally, paraffin-embedded sections can be used for immunohistochemistry and immunofluorescence and are compatible with multiplexing staining, which uses multiple immunostains on the same tissue section, as well as computational 3D reconstruction techniques using a series of tissue sections [11].

## Materials and reagents

**Biological materials**

1. Organoids (300 μm to 3 mm in size) embedded in matrix domes

**Reagents**

1. Double-deionized water produced by the Synergy water purification system and ion exchange resin water system (Merck Millipore, catalog number: SYNSVHFFR-KIT and Veolia water system, catalog number: CFCFGU201181)

2. Methanol-stabilized formaldehyde 30% in solution (Carl ROTH, catalog number: 4235)

3. Sodium azide (NaN_3_) (Sigma-Aldrich, catalog number: S8032)

4. Potassium chloride (KCl) (Sigma-Aldrich, catalog number: P5405)

5. Potassium dihydrogen phosphate monobasic (KH_2_PO_4_) (Sigma-Aldrich, catalog number: P0662)

6. Sodium chloride (NaCl) (Sigma-Aldrich, catalog number: S7653)

7. Sodium phosphate dibasic heptahydrate (Na_2_HPO_4_·7H_2_O) (Sigma-Aldrich, catalog number: S9390)

8. Glycine, aminoacetic acid (Sigma-Aldrich, catalog number: G8898)

9. Butanol-2-Methyl-1-butanol (Sigma-Aldrich, catalog number: 133051-100ML)

10. Xylene histological grade [C_6_H_4_(CH_3_)_2_] (Sigma-Aldrich, catalog number: 534056-500ML)

11. Absolute ethanol (CH_3_CH_2_OH) (Sigma-Aldrich, catalog number: 1070172511); when necessary, dilute ethanol with double-deionized water

12. Epredia HistoGel specimen processing gel (Epredia, catalog number: HG4000012)

13. Epredia Paraffin Type 9 (Epredia, catalog number: 8337)

14. Hematoxylin GILL II (Diapath, catalog number: CP813)

15. Trichloroacetic acid (Thermo Fisher Scientific, catalog number: 7402)

16. Lithium carbonate (Microm Microtech, catalog number: 45006 29)

17. Eosin (Diapath, catalog number: C0362)

18. Saffron (VWR, catalog number: 27481.105)

19. Diamount mounting medium (Diapath, catalog number: 030400)

20. Dako Target retrieval solution, citrate pH 6.1, 10× (Agilent, catalog number: S1699)

21. ReadyProbes hydrophobic barrier pap pen (Life Technologies, catalog number: R3777)

22. ProLong Gold anti-attenuation mounting medium (Life Technologies, catalog number: P36930)

23. Dako protein block solution, ready to use (Agilent, catalog number: XO909)

24. Dako antibody diluent, ready to use (Agilent, catalog number: SO809)

25. Antibody (depending on application)

**Solutions**

1. PBS 10× (see Recipes)

2. PBS/glycine (see Recipes)

3. 2% formaldehyde (see Recipes)

**Recipes**

**1. PBS 10×**

**Table:** 

Reagent	Final concentration (g/L)	Final concentration (mM or M)	Quantity or volume
Double-deionized water	1×	n/a	1,000 mL
Potassium chloride	2 g/L	26.8 mM	2 g
Potassium dihydrogen phosphate monobasic	2 g/L	14.7 mM	2 g
Sodium chloride	80 g/L	1,369 M	80 g
Sodium phosphate dibasic heptahydrate	21.6 g/L	80.6 mM	21.6 g

Dilute 10× solution with double-deionized water for 1× utilization. Store at 4 °C for up to one year.

**2. PBS/glycine**

**Table:** 

Reagent	Final concentration (g/L)	Final concentration (μM or M)	Quantity or volume
PBS 10×	10×	n/a	1,000 mL
Glycine	75 g/L	1 M	75 g

Dilute 10× solution with double-deionized water for 1× utilization. Store at 4 °C for up to six months.

**3. 2% Formaldehyde**

**Table:** 

Reagent	Initial concentration	Final concentration	Quantity or volume
PBS	10×	1×	6 mL
Double-deionized water	1×	1×	50 mL
Formaldehyde (methanol-stabilized)	30%	2%	4 mL

Prepare fresh before use.

**Laboratory supplies**

1. Complete staining sets: well, cover, slide rack (Epredia, catalog number: 121)

2. Slides for microscopy Epredia SuperFrost Plus Gold (Epredia, catalog number: K5800AMNZ72)

3. Lid of 48-wells plate (Corning, catalog number: 3548)

4. Immunostaining slide moisture chambers (Evergreen, catalog number: 240-9020-Z10)

5. Dissection Fine-Pointed Forceps (Fisherbrand, catalog number: 08-880)

6. 660 mL glass jar (Dutscher, catalog number: PI82PC660)

7. White Pasteurizable lid, TO 82 ring (Dutscher, catalog number: CPAST82BL)

8. Razor blade compatible with microtome (ThermoFisher Scientific, catalog number: 3050835)

9. 12-piece blue HB pencil set (Lyreco, catalog number: 013LYRK7720/1)

10. Epredia Cryotom Cryostat Accessories, Camel HairBrush (Epredia, catalog number: 1910)

11. Epredia Shandon disposable embedding molds 15 × 15 mm (Epredia, catalog number: 41741)

12. Epredia Shandon disposable embedding molds 24 × 30 mm (Epredia, catalog number: 41743)

13. Fisherbrand graduated transfer pipettes, non-sterile, LDPE, clear (Fisher Scientific, catalog number: 13439108)

14. Epredia Cassette II slotted tissue cassettes (Epredia, catalog number: B851729)

15. Biopsy foam pads (Simport Scientific, catalog number: M476-1)

## Equipment

1. 115 V Programmable Dry Bath Incubator (Boekel Scientific, catalog number: 115001)

2. Paraffin dispenser (Bio-Optica Milano, catalog number: 40-200-101)

3. Cooling plate (Micro Microtech, catalog number: EC 350-2)

4. Decloaking Chamber^TM^ NxGen (Biocare Medical, catalog number: DC2012-220V)

5. Block Module, 16 mm tubes (Boekel Scientific, catalog number: 110016)

6. Stuart Mini Gyro-Rocker SSM3 (Fisher Scientific, catalog number: 10034264)

7. Microtome Microm (Epredia, catalog number: HM355S)

8. Epredia^TM^ STP 120 Spin Tissue Processor (Epredia, catalog number: STP 120)

9. Automated slide stainer Gemini AS (Epredia, catalog number: A81500001)

10. Epifluorescence microscopes (Zeiss, Axio Observer 7 with LED Colibri 7 and HAMAMATSU C11440 digital camera; Nikon, Eclipse Ti2 with DS-Fi3 brightfield camera and DS-Qi2 fluorescence camera)

## Procedure

**A. Sample preparation**

1. Sample fixation

a. Remove culture media and add 2% formaldehyde solution (see Recipe 3) to fix the organoids directly in matrix domes.

b. Incubate for 90 min at room temperature under slow rocking agitation (between 20 and 25 rpm).

*Note: A 90-min incubation ensures that formaldehyde efficiently penetrates organoids ranging from 500 μm to 2 mm, ensuring uniform fixation. The fixation time can be adjusted depending on organoid size.*

c. Remove 2% formaldehyde and wash three times for 10 min with PBS/glycine (see Recipe 2).

d. Store at 4 °C in PBS with azide 0.1% to prevent microbiological contamination.

*Note: PBS with 0.1% azide can be prepared in advance and stored at room temperature for 6 months. Sodium azide is highly toxic and requires the use of proper personal protective equipment. Sodium azide needs to be disposed of in a proper container according to country regulations.*

**Pause point:** Fixed organoids can be stored at 4 °C for 2 months (or 12 months if you add PBS with 0.1% azide every 2 months to prevent drying out the fixed samples).

*Note: We recommend including organoids in paraffin soon after fixation to avoid tissue degradation over time.*

**Figure 1.**
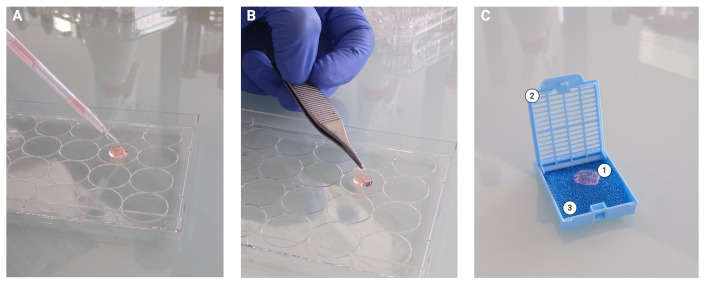
Setup of HistoGel inclusion. (A) A first droplet of HistoGel is deposited on the cover of a 24-well plate. (B) Organoids in Matrigel domes are delicately deposited on top of the HistoGel droplet. (C) Representative image of organoids included in HistoGel (1), inside a histological cassette (2), and covered with foam (3).

2. Preparation of fixed samples before inclusion

a. Heat the HistoGel for 30 min at 90 °C with a block module.

b. Make 100 and 250 µL drops of warm liquid HistoGel using transfer pipettes on a 48-well plate lid (Figure 1; Video 1).

c. Gently detach the matrix domes containing the organoids with a metal clip and place them in the HistoGel drop.

*Notes:*

*1. Alternatively, put the organoids directly in the HistoGel drops if you culture your 3D organoids without Matrigel, matrix, or polymer.*

*2. It is possible to put 2 or 3 matrix domes (each with several organoids) inside one HistoGel drop. This is recommended when the organoid yield per dome is low, e.g., 10 organoids per dome.*

d. Wait for 5 min for the HistoGel to cool, then place another droplet of HistoGel on top of each organoid dome.

e. Wait for 30 min for the HistoGel to polymerize and then remove any excess product using the flat side of microdissection forceps or a scalpel.

f. Place the dome containing organoids on a cassette that has been previously covered with biopsy foam.

*Note: Adjust the number of domes loaded into each cassette according to the total number of organoids available and the desired representation in the final tissue sections. When organoid yield per matrix dome is low, multiple domes may be embedded within the same cassette to increase the likelihood of interesting organoids during microtomy (up to 4 domes per cassette, or up to 8 depending on mold dimensions). Under standard conditions, 1–2 domes are typically placed in each histology cassette.*

g. Close and submerge the cassettes in 70% ethanol overnight at 4 °C.

**B. Sample inclusion**

1. Dehydration of samples

a. Perform the dehydration of organoids using the Spin Tissue Processor with the following program:

Ethanol 80% 10 min

Ethanol 95% 10 min

Ethanol 95% 10 min

Ethanol 95% 10 min

Ethanol 100% 10 min

Ethanol 100% 10 min

Ethanol 100% 10 min

Butanol 15 min

Butanol 15 min

Butanol 15 min

Paraffin wax 40 min

Paraffin wax 40 min

*Notes:*

*1. Butanol is used here because it allows a gentler dehydration than ethanol or methanol. It reduces tissue shrinkage, which is recommended for fragile tissues such as organoids.*

*2. The last two paraffin cycles ensure complete and deep penetration of paraffin into Matrigel and organoids. This decreases the risk of ribbon breakage during section processing.*

*3. Switch on the paraffin dispenser to prepare for the next step and have the melted paraffin ready by the end of the inclusion cycle. Also, turn on the cooling plate approximately 30 min before the next step.*

2. Inclusion

a. Transfer the cassettes into the paraffin fountain.

b. Pour liquid paraffin wax into the bottom of a disposable embedding mold.

c. Wait a few seconds above the cooling station for the paraffin to harden.

d. Remove the cassette from the paraffin fountain and transfer the dehydrated HistoGel drops to the base mold.

*Note: Incorporate 2–3 HistoGel droplets into the same paraffin block to increase the number of organoids per slide.*

e. Place paraffin wax in the mold to fill it.

f. Remove the cassette lid and place the bottom part on top of the mold to create a tissue block.

g. Let the paraffin harden on the cooling station for 15–30 min.

h. Remove the disposable base mold from the paraffin tissue block.

**Pause point:** Paraffin blocks can be stored at room temperature until sectioning.

**C. Sample block section**

1. Turn on the microtome, position the paraffin block, and secure it with the screws.

2. Bring the paraffin block near the razor blade and lower the blade protection.

*Note: The razor blade should be changed at the end of each sectioning session or when the quality of the sliced paraffin ribbon decreases.*

3. Align the paraffin block parallel to the razor blade.

4. Select cutting speed and thickness and then cut the tissue into strips.

*Notes:*

*1. We recommend first trying a speed between 10 and 15 mm/s, with a thickness of 5 μm.*

*2. If the sections crumble, rehydrate the blocks by soaking them in a PBS bath on ice for at least 30 min.*

5. Use a paintbrush to gather the paraffin sections and gently float them in a bath of distilled water warmed to 35 °C ± 5 °C.

*Note: If the bath temperature exceeds 45 °C, the paraffin might melt again.*

6. Place histological sections on a slide and allow them to dry for a few minutes at room temperature.

*Note: Slides can be kept in a slide box at room temperature until they are needed again.*

**D. Sample immunostaining**

**D1. Dewaxing**

1. Incubate the slide for 1 h at 60 °C in a dry oven and proceed to dewax by immersing it in two successive 15-min xylene baths.

2. Rehydrate slides by immersing them sequentially in the following series of baths:

100% ethanol for 5 min

95% ethanol for 5 min

85% ethanol for 5 min

70% ethanol for 5 min

Deionized water 2 times for 2 min

*Note: Once the procedure is complete, do not leave the slides in deionized water or let them dry out, as this can cause the tissue to degrade. Proceed immediately to slide coloration or immunofluorescence staining.*

**D2. Hematoxylin and eosin coloration**

1. H&E staining can be performed using an automated slide stainer. The staining procedure can then be carried out as follows:

Hematoxylin 3 min

Deionized water 3 min

Trichloroacetic acid 45 s

Deionized water 1 min

Lithium carbonate 20 s

Deionized water 1 min

Eosin 6 min

Deionized water 1 min

Ethanol 100% 2 baths

Saffron 2 min

2. Proceed to slide dehydration by bathing them in the following series of baths:

70% ethanol for 5 min

85% ethanol for 5 min

100% ethanol for 5 min

Xylene for 5 min

Xylene for 5 min

Mount slides with Diamount mounting medium or other non-aqueous mounting medium.

**D3. Immunofluorescence staining**

1. Antigen unmasking

**Figure 2.**
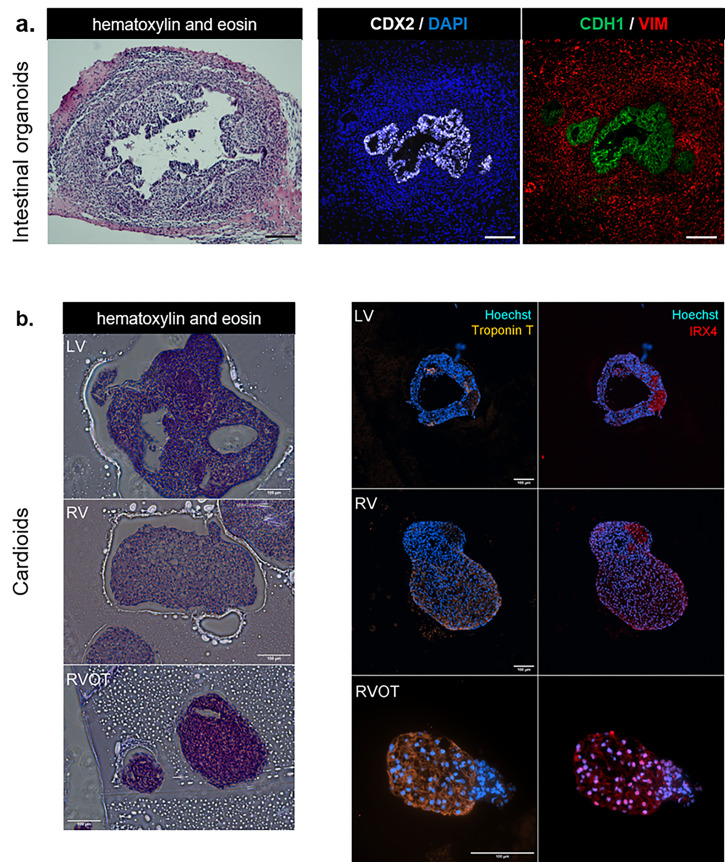
Histology and immunostaining of organoids. (A) Hematoxylin and eosin (H&E) staining (left) and immunofluorescence staining (right) of paraffin-embedded human intestinal organoid sections. Human intestinal organoids are characterized by expression of the hindgut marker CDX2, the epithelial marker Cadherin-1 (CDH1), and the mesenchymal marker Vimentin (VIM). Scale bars: 100 μm. Representative images of >20 biological replicates. (B) H&E staining (left) and immunofluorescence staining (right) of paraffin-embedded human cardioid sections. Left ventricle (LV), right ventricle (RV), and right ventricular outflow tract (RVOT) are stained for the cardiomyocyte marker Troponin T and the ventricular myocytes IRX4. Scale bars: 100 μm. Representative images of >10 biological replicates.

**Critical:** Paraffin-embedded tissues typically require an antigen unmasking step before staining to ensure optimal epitope–antibody binding. Various antigen retrieval techniques and solutions are available and should be selected based on the target antigen and the antibody used. The following steps outline the antigen unmasking procedure used to achieve the stainings shown in Figure 2.

a. Dilute 20 mL of Dako Target retrieval solution pH 6 into 180 mL of distilled water.

**Caution:** Use the Dako retrieval under a hood suitable for CMR substances.

*Note: Dako retrieval solution also exists at pH 9. Make a choice based on the antibodies used and their data sheet.*

b. Submerge the slide into the Dako retrieval solution and perform heat-induced epitope retrieval in a decloaking chamber at 110 °C for 90 s.

*Note: Pressure parameters cannot be set for the decloaking chamber, but this device reaches 25 psi at 110 °C.*

c. Discard the Dako Target retrieval solution once it has cooled. Then, rinse the slides with three consecutive PBS baths.

2. Antibody staining

a. Outline tissue sections using a hydrophobic pap pen to help contain reagents.

b. Incubate slides with protein block solution for 1 h in a humidity chamber at room temperature.

c. Aspirate blocking solution.

d. Incubate slides with primary antibodies diluted in Dako protein block solution at 4 °C overnight in a slide moisture chamber.

*Note: Follow the antibody datasheet to achieve the most appropriate dilution for the tissue analyzed. Typically, for organoid staining, the dilution range is 1:200–1:1,000, depending on the manufacturer’s instructions.*

e. Wash slides in PBS for 10 min with gentle agitation. Repeat the procedure three times.

*Note: We advise against washing slides in racks to avoid antibody cross-contamination and to prevent tissue damage or loss during the wash.*

f. Incubate slides with secondary antibodies diluted in Dako protein block solution for 1 h at room temperature in a slide moisture chamber.

*Note: Follow the antibody datasheet to achieve the most appropriate dilution for the tissue analyzed.*

g. Wash the slides in PBS for 10 min, protecting them from light. Repeat this process three times.

h. Incubate slides with 1 μg/mL of DAPI in PBS for 5 min while protected from light.

i. Wash microscope slides in PBS for 3 × 10 min each, while protected from light.

j. Mount microscope slides with coverslips using Prolong Gold mounting medium or another aqueous mounting medium.

**Critical:** Remove any bubbles that have a refractive index different from that of the mounting medium.

k. Proceed with imaging under an epifluorescence microscope.

**Video 1.**
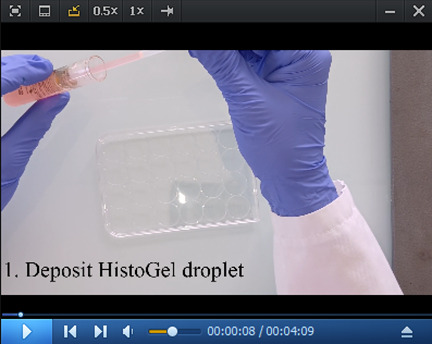
Inclusion of organoids in HistoGel

## Validation of protocol

This protocol has been used and validated in the following research articles:

- Vales S, Bonneau L, Mahe MM. Differentiating Enteric Glial Cells Within Intestinal Organoids Derived from Human Pluripotent Stem Cells. *Methods Mol Biol*. (Figure 3)
- Bonneau L et al. Generation of intestinal and colonic organoids derived from human pluripotent stem cells. *Biology of the Cell*. (Figures 2b and 3a)

## General notes and troubleshooting

**Troubleshooting**

**Problem 1:** HistoGel drops shrank after dehydration.

Possible cause: The HistoGel has expired or undergone too many liquefaction cycles (after three liquefaction cycles, the quality of HistoGel cannot be guaranteed).

Solution: Use a fresh tube of HistoGel or a new batch.

**Problem 2:** Paraffin sections break apart and disintegrate during sectioning.

Possible causes: The room is too hot, the paraffin block is too dry, or the embedding has failed.

Solution: Before sectioning, rehydrate the block by immersing it in PBS at 4 °C for 30 min, or melt the block and re-embed the samples (section B).

**Problem 3:** Samples peel off the slides after dewaxing by rehydration.

Possible cause: The section is of poor quality, or the blades used are not recommended for this type of experiment.

Solution: Perform new tissue sections and ensure the use of unused, certified blades.

**Problem 4:** Antigen retrieval is uneven.

Possible cause: The parameters of the decloaking chambers do not match the antibodies used.

Solutions: Adjust the temperature, time, and pressure of the decloaking chamber. Use a Dako Target retrieval solution at a different pH that better suits the antibody, following the manufacturer’s instructions.

**Problem 5:** Immunofluorescence signal is weak and/or has excessive background fluorescence.

Possible causes: The antibody is too diluted, not specific, or has expired.

Solutions: Try to increase the concentration of the antibody (1:200 or 1:100); alternatively, use a new batch.

**Problem 6:** During the tissue sectioning process, too many slices are empty, lacking biological material.

Possible cause: The Matrigel droplet did not contain enough organoids.

Solution: Increase the number of HistoGel droplets included in the same histological cassette. If matrix domes contain very few organoids, include up to 4 droplets per histological cassette. This increases the chance of cutting through biological material during sectioning.
